# *Ex situ* conservation of *Ruscus aculeatus* L. – ruscogenin biosynthesis, genome-size stability and propagation traits of tissue-cultured clones

**DOI:** 10.1080/13102818.2014.984976

**Published:** 2015-01-08

**Authors:** Teodora Ivanova, Dessislava Dimitrova, Chavdar Gussev, Yulia Bosseva, Tatyana Stoeva

**Affiliations:** ^a^ Department of Plant and Fungal Diversity and Resources, Institute of Biodiversity and Ecosystem Research, Bulgarian Academy of Sciences, Sofia, Bulgaria

**Keywords:** cytometry, HPLC, micropropagation, origin, *in vitro*

## Abstract

*Ruscus aculeatus* L. is a perennial semi-shrub with distinctive leaf-like branches (cladodes). Rhizomes and roots contain steroidal saponins (ruscogenins) that are used in medicine and cosmetics for their anti-inflammatory, venotonic and antihaemorroidal activity. Problematic cultivation of the species causes in many countries unsustainable over-collection from the wild. Tissue culture propagation of *R. aculeatus* was carried out for conservation and propagation purposes. The impact of the clonal origin (genotype) on the ruscogenin biosynthesis, genome-size stability and propagation traits and morpho-physiological response to long-term cultivation *in vitro* was studied. Production of ruscogenins in fully developed regenerants was quantified by high-performance liquid chromatography (HPLC). Genome-size stability of the clones was assessed by flow cytometry. Slow growth and prolonged lag-phase were characteristic for the whole propagation cycle. Produced plantlets with well-defined organs were suitable for direct *ex vitro* planting. Genome DNA content of all clones was stable and comparable to native plants. Ruscogenin biosynthesis was clone-specific, presenting distinctive profiles of the cultures. Our results imply that clone origin and culture type might influence saponin biosynthesis in *Ruscus.* These traits should be considered in the *ex situ* conservation of the genetic diversity of this species and by production of planting material as well.

## Introduction

*In vitro* techniques are a powerful tool for *ex situ* plant conservation. They offer convenient storage in small space and controlled environment for valuable plant species that are hampered to propagate by seeds or by vegetative material. Adequate conservation practices require verification of the genetic conformity and normal physiology of the obtained plants.[1,2] Clonal origin (genotype) is considered as a crucial factor for the culture performance in many species. Its impact is very important especially for conservation programmes since the protocols are generally based on one or few donor individuals. The effectiveness could be limited by species- and clone-specific traits causing recalcitrance of the cultures.[3] The *in vitro* storage procedure itself can cause unnatural variation or occurrence of rare variants, especially when dedifferentiation stage is used (reviewed in [4–9]).

*Ruscus aculeatus* L. (*Ruscaceae*) is a slowly growing perennial semi-shrub, naturally distributed in the Mediterranean, South Central Europe, Caucasus and Crimnea.[10] *Ruscus aculeatus* is introduced as a garden and house plant in Britain and North America, as the evergreen stems with typical leaf-like flattened branches (cladodes) are prized as ornamental greenery. Rhizomes and roots contain steroidal saponins (ruscogenins) that are used in medicine and cosmetics for their anti-inflammatory, venotonic, antihaemorroidal activity.[11–14] The herbage, as greenery, and the pharmaceutical raw material are collected mainly from the wild.[15,16] Complicated cultivation of *Ruscus* species causes their unsustainable over-collection from the natural populations and management of wild resources remains an unresolved issue in some countries.[7,17–19] Slow plant growth and development associated with limited growth of the aboveground organs and pollination failure makes the species vulnerable to distress in the natural population.[20] Conservation efforts, so far, have relied on limitation or prohibition of gathering together with cultivation trials with partial success.[18,21,22] *In vitro* cultivation of *R. aculeatus* was carried out to meet the limitations of the conventional propagation (rhizome splitting). Research teams, mostly, in Mediterranean and Balkan countries have developed propagation protocols with donor material from wild populations, but none has reached commercial stage yet.[23–26] Presented results are often partial, presenting different gaps in the initiation or propagation stages. Problems were related to decontamination, slow growth and lack of shoot regeneration. However, the limited data does not allow defining the production ‘bottle-necks’.

This work evaluates significance of the clonal origin on performance of the *R. aculeatus* cultures by assessment of the ruscogenin biosynthesis, genome-size stability, propagation rate and morpho-physiological response to long-term cultivation *in vitro*.

## Materials and methods

### Plant material and culture conditions

Seeds were collected from sites in Strandzha Mt. (SE Bulgaria)–A (1–3) and Stara Planina Mt. (West Bulgaria)–B (1–4) as part of the Millennium Seed Bank Partnership collection missions. Voucher specimens were deposited in the Herbarium of Institute of Biodiversity and Ecosystem Research (SOM). Shoot cultures were initiated from rhizome segments of seedlings by a previously described technique.[27]

Seven seed-derived clones from Bulgarian wild populations were compared. Cultures were maintained continuously on Murashige & Skoog (MS) media [28] with 1 mg/L 6-Benzylaminopurine (BAP), 0.5 mg/L α-Naphthaleneacetic acid (NAA), 0.8 mg/L Plantagar (all supplied by Duchefa, Netherlands) and 30 g mg/L commercial sugar. All media were adjusted to pH 5.75 prior to sterilization and autoclaved at 121 °C/1 atm for 20 minutes. The cultures were maintained in VitroVent® containers (with 125 mL medium, at 16/8 h photoperiod and 23 ± 1 °C). Cultures were transferred onto fresh media every eight weeks and every shoot with minimal height of 0.5 mm was detached and grown separately on the same medium. Propagation rate, shoot growth and appearance were recorded for at least 18 months.

### Genome-size stability

DNA content of the cultures from all clones was measured using flow cytometry with propidium iodide staining, using Partec CyFlowR SL and following the protocol provided by Partec (Sysmex Partec GmbH, Germany). *Pisum sativum* cv. *Kleine Rheinländerin* (2*С* = 8.84 pg) was used as internal standard. Native *R. aculeatus* plants from Stara Planina Mt. were used as reference control. Fresh cladode samples were collected and genome size was measured in three replicates, each run with 5000 counts. Data were analysed statistically for variation using Duncan's multiple range test.

### Ruscogenin quantification

Production of ruscogenins in shoots, rhizomes and roots of fully developed regenerants was determined by HPLC following adapted procedure.[29] Fine powdered samples (50 mg) of at least 50 fully developed *in vitro* plants were prepared by drying at 35 °С for 48 h. Extraction was done twice with 4 mL 50:50 aqueous methanol for 24 h, followed by evaporation under vacuum (Heidolph Laborota 4003 Rotary Evaporator, at 40 °С). Dry extracts were hydrolyzed for 4 h at 80 °C with 4mL *n*-butanol, 1 mL deionized water and 0.55 mL 37% HCl. Samples were neutralized with 5% NaHCO3 solution and washed. Butanolic fractions were filtered (0.20 μm PTFE syringe filters, DISMIC, Denmark) and evaporated to dryness. Residues were dissolved in 200 μL methanol (GC-grade, Leda) and filtered (Waters syringe filters, 0.45 μm, PTFE) before loading in autosampler vials (Waters 717plus). Chromatographic system: column: Phenomenex Synergi MAX-RP 80A 4 μm, 150 × 4.6 mm; pre-column: Synergi MAX 4 × 3.0 mm; detector: Waters M 996 Diode Array Detector PDA Max plot 180–800 nm; pump: Waters 600E. Chromatographic conditions: injection volume = 10 μL; *V* = 1 ml/ml, *T* = 25 °C. Elution gradient: А (acetonitrile:water) 65:35; B (acetonitrile:water) 50:50 (Table 1).

**Table 1.** Elution conditions for ruscogenin HPLC quantification.

Min.	A	B	Curve
0	0	100	
7.5	0	100	1
13	100	0	8
20	100	0	6
22	0	100	11
24	0	100	11

Quantification was done against standards of the sapogenins, ruscogenin and neoruscogenin and two desmosides of the neoruscogenin:ruscin and desglucoruscin (butchers broom *Ruscus aculeatus* root VBRM ChromaDex®, USA). Calibration curves were set between 0.2 and 4 μg/0.01 mL.

## Results and discussion

**Figure 1.**
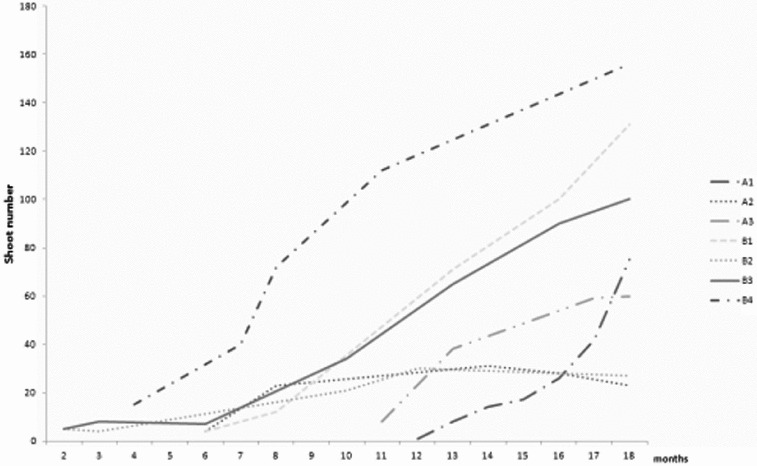
Propagation rate of *R. aculeatus* clones for 18 months. A (1–3): seed-derived clones from Strandzha Mt.; B: seed-derived clones from Stara Planina Mt.

Dormancy in *R. aculeatus* seeds is considered to be morpho-physiological and spans between 2 months and year and a half.[10,30] Germination rates were described as variable depending on the seed lot, age and origin of the seeds.[23,26,31] Seedlings had short rhizomatous stem and usually one shoot. Slow growth was characteristic for all *R. aculeatus* clones (Figure 1). Seedlings presented a rather variable mode of growth and performance under controlled conditions. In the propagation stage, new shoots were induced directly on the surface of the rhizome explants in the limited proliferative area around the apical and adventitious buds. Only three of the tested clones showed propagation rates above 100 regenerants over the period of 18 months. All clones produced shoots with upright aerial stem, rarely branched, having 3–11 elliptical cladodes. Cultures tended to develop rooted shoots suitable for direct *ex vitro* planting. Limited shoot number could be related to apical dominance, suppressing new shoot development as shown in other rhizomatous species.[32–34] Nevertheless, prolonged initiation phase and reduced shoot number was reported for *R. aculeatus*, even by authors using callus cultures.[23,25] Delayed regeneration in the closely related *Asparagus officinalis* is associated with continual alternation in sensitivity to growth regulators.[35] Similar tendency was distinctive within three years of *R. aculeatus* cultivation. Raise in the propagation rate was linked to changed sensitivity to BAP in the media (data not shown). This habit was considered as positive for longer *ex situ* storage as most of aged cultures tend to worsen their vitality with time.[2,36] Differential requirement of growth regulators due to genotypic differences was shown as an important factor for micropropagation of *Curcuma* cultivars.[37–39]

**Figure 2.**
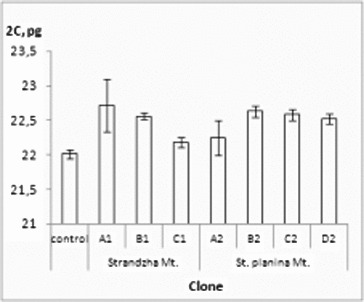
Genome size of the *R. aculeatus in vitro* clones. Values in pg ± SE. Control – native *R. aculeatus* plants. Internal standard – *P. sativum*.

Flow cytometry is one of the convenient and quick methods to test genome stability of *in vitro* obtained plants and was readily applied to variety of species in the recent years.[41–43] Present evaluation confirmed genome-size stability of all clones (Figure 2). 2*C*-values ranged from 22.00 to 22.71 pg. The average genome DNA (2*C* = 22.49 pg) was slightly higher than the native control and close to previously reported values.[40] Statistically significant differences were not observed.

**Figure 3.**
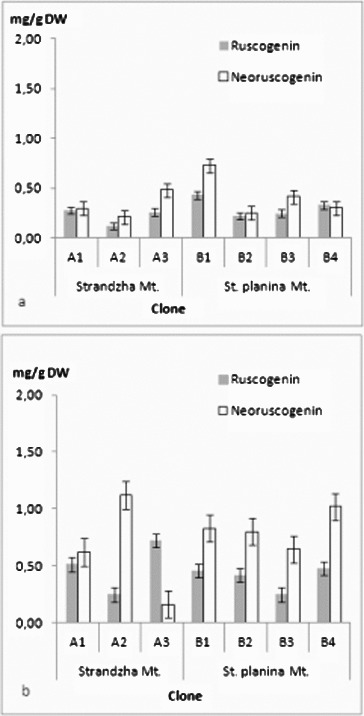
Ruscogenin and neoruscogenin content in (a) shoots and (b) rhizomes and roots. Values in mg/g DW ± SE.

**Figure 4.**
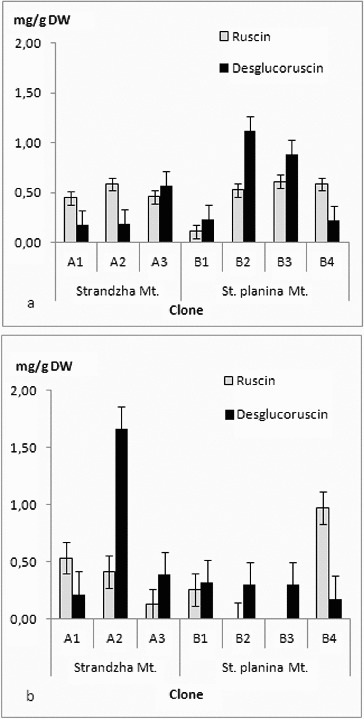
Ruscin and ndesglucoruscin content in (a) shoots and (b) rhizomes and roots. Values in mg/g DW ± SE.

The four ruscogenins were determined in all *in vitro* clones (Figures 3(a) and 3(b) and 4(a) and 4(b)). There was no clear tendency for grouping by geographical basis. Even more every clone had its own specific profile. Current ratios for ruscogenin and neoruscogenin were similar to those reported for Bulgarian populations.[44] Quantitatively contents were similar to those reported for Romanian callus-derived clones.[45] Highest amounts in shoots were recorded for the clone B1 – neoruscogenin (0.73 mg/g DW) and ruscogenin (0.43 mg/g DW). These were about the average for the rhizome and roots, where most productive for neoruscogenin and ruscogenin were clones A2 and A3, respectively (1.12 mg/g DW and 0.71 mg/g DW). All clones had about twice time lower production of ruscogenin than neoruscogenin in the underground organs. *In vitro* (callus) cultures were reported to have limited biosynthetic abilities with increase of the neoruscogenin and ruscogenin contents following the organogenesis.[29] Comparing all measured ruscogenins, it was evident that roots were more productive than shoots, contrary to *in vitro* cultures of Spanish origin.[46] Neoruscogenin and ruscogenin in cladodes were mostly even. Neoruscogenin prevailed in rhizomes and roots. Only one of the clones (A3) showed ruscogenin domination. Studies in natural populations in Turkey and Romania revealed that neoruscogenin was higher in the shoots rather than in the roots, and for the ruscogenin the organ distribution was contrasting.[28,47] Ruscin and desglucoruscin were measured for the first time in tissue cultures, performing considerable amounts both in shoots and underground parts, similar to neoruscogenin. Desglucoruscin reached highest value in rhizomes and roots of clone A2 (1.66 mg/g DW) and for the shoots in clone B2 (1.12 mg/g DW). Ruscin was missing in underground parts of B2 and B3 clones. These results suggested that origin of the material on population as well as on individual level could be important factor for the saponin biosynthesis. Moreover, the obtained data does not support the hypothesis that ruscogenin biosynthesis occurs mainly in the aerial shoots.[46] The differences could be related not only to genotype but also to culture type and conditions, explants source, regenerants age and sampling time, as the yield of secondary metabolites depends on multiple factors.[48–50] The obtained results are particularly interesting, considering the limited number of seed collections of *R. aculeatus*,[51] low germination rates [21] and the recalcitrant micropropagation habit reported previously.[23,25,27]

## Conclusions

Considering the above-mentioned issues recovery of *R. aculeatus* populations and production of planting material by micropropagation could be greatly influenced by the choice of the source material. However, *in vitro* preservation is suitable for conservation of the species with minimal efforts as flow cytometric data confirm the stability of the genome size of the regenerants. Still clone-specific propagation rates and ruscogenin profiles necessitate large number of donor plants to be maintained so as to cover the genetic diversity of the species.

## Funding

This work was supported by the Bulgarian Ministry of Education and Science [grant number BG051PO001/07/3.3-02/70].
